# Evaluation of the Effects of Smoking on Trabecular Bone Microarchitecture Using Cone-Beam Computed Tomography in Periodontal Disease

**DOI:** 10.7759/cureus.98851

**Published:** 2025-12-09

**Authors:** Sibel Kayaaltı Yuksek, Cansu Buyuk, Ipek Cebeci, Gonca Cayir Keles

**Affiliations:** 1 Department of Periodontology, Faculty of Dentistry, Istanbul Okan University, Istanbul, TUR; 2 Department of Oral and Maxillofacial Radiology, Faculty of Dentistry, Istanbul Okan University, Istanbul, TUR

**Keywords:** bone-morphometric parameters, cone-beam computed tomography, periodontitis, smoking, trabecular bone

## Abstract

Objectives: The quality and structure of alveolar bone play a critical role in periodontal health and disease. Tobacco use has long been associated with negative impacts on bone and periodontal tissues. This cross-sectional study aimed to determine whether smoking induces quantifiable deterioration in trabecular bone microarchitecture in individuals with periodontal disease through bone morphometric analysis of cone-beam computed tomography (CBCT) images.

Method and materials: Ninety systemically healthy patients, comprising both smokers (≥10 cigarettes/day) and non-smokers who required CBCT imaging for various indications, were included. Clinical periodontal parameters (plaque index, gingival index, probing pocket depth, clinical attachment loss (CAL), bleeding on probing) were recorded. Participants were grouped by smoking status and subdivided by periodontal diagnosis: gingivitis, mild (stages I and II), moderate, and severe (stages III and IV) periodontitis (n = 15 per subgroup). Mandibular first molars and central incisors were selected without restorations, endodontic lesions, or root canal treatments. Two 5 × 5 × 10 mm volumetric regions of interest (ROIs) were analyzed around the periapical regions of related teeth: posterior and anterior ROIs (180 ROIs). Trabecular bone microarchitecture was examined using the BoneJ plugin of ImageJ software (National Institutes of Health, Bethesda, Maryland) to measure the bone morphogenic parameters (BMPs): degree of anisotropy (DA), bone volume fraction (BV/TV), trabecular thickness (Tb.Th), and trabecular separation (Tb.Sp). Statistical analysis was conducted using the Student's t-test, one-way ANOVA, and Kruskal-Wallis tests (Clinical Trial: NCT06676358).

Results: Differences in BMPs were observed between groups, and correlation was identified between age, gender, and BMPs (p > 0.05). However, a significant correlation was found between posterior BV/TV and CAL (p < 0.05), and an increasing trend in Tb.Th with higher attachment loss of the related first molar was noted in the posterior ROI.

Conclusion: While periodontal status and smoking did not affect BMPs, posterior BV/TV was associated with CAL in periodontitis patients.

## Introduction

Alveolar bone is a dynamic and actively renewing tissue influenced by various factors, including mechanical forces, inflammation from periodontal disease, hormonal regulation, age-related changes, medications, smoking, and systemic diseases such as diabetes and rheumatoid arthritis. In healthy periodontium, a balance between bone resorption by osteoclasts and bone formation by osteoblasts is essential. Periodontitis, characterized by bacteria-induced chronic inflammation and bone loss, disrupts this balance and highlights the intricate relationship between inflammation and bone loss [1, 2].

The inflammatory response can interfere with bone formation through multiple mechanisms. Inflammation can disrupt bone formation through increased apoptosis in bone-lining cells, reducing bone-forming cells, cytokines inhibiting osteoblast differentiation, and decreased bone matrix production. These processes reduce osteoblastic cell numbers, preventing an adequate compensatory formation of new bone for resorbed areas and ultimately contributing to an imbalance and further periodontal bone loss [3]. Also, osteoclasts activated by inflammatory signals play a pivotal role in bone resorption. Osteoclast differentiation and activation are primarily regulated by the RANK/RANKL signaling pathway, where RANKL, produced by osteoblasts and other cells in response to inflammation, binds to RANK on osteoclast precursors to promote their maturation. In periodontal disease, bacterial products such as lipopolysaccharides trigger an inflammatory response, leading to the release of pro-inflammatory cytokines such as TNF-α, IL-1, and IL-6 that enhance RANKL expression and stimulate osteoclastogenesis. Osteoclast activation disrupts the balance between bone formation and resorption, contributing to increased periodontal bone loss [4].

Smoking can contribute to inflammatory diseases through mechanisms such as genetic modifications, oxidative stress, and the production of free radicals. These processes lead to increased proliferation of B and T cells, reduced activity of immunosuppressive T regulatory cells, and elevated levels of pro-inflammatory mediators, including IL-1β, IL-6, and TNFs [5]. The resulting chronic inflammatory state promotes osteoclastogenesis and inhibits osteoblast function, disrupting the balance between bone resorption and formation [6]. A meta-analysis indicated that heavy smoking, defined by the duration and quantity of cigarettes smoked, correlates with reduced bone mineral density [7]. In addition, smoking affects periodontal health by disrupting the microbiological, immune-inflammatory, and physiological responses to periodontal disease. Smokers face a significantly higher risk of developing periodontitis and alveolar bone loss, with studies showing a four times increased likelihood compared to non-smokers [8, 9].

CBCT imaging has become widely adopted in dentistry for detailed imaging of dental and surrounding structures [10]. Within trabecular bone, trabeculae are arranged in various directions, thicknesses, and porosity levels, contributing to the structural stiffness of the bone. The microarchitecture of trabecular bone is an important indicator for assessing the mechanical properties of the bone [11]. Indicators used to assess bone quality include the DA, BV/TV, Tb.Th, and Tb.Sp metrics. BV/TV and Tb.Th reflect the bone's capacity to resist mechanical loads, while DA reveals trabecular orientation, and Tb.Sp indicates the spacing between trabeculae, impacting overall density and structural stability [12]. CBCT-based assessments of bone quality have demonstrated sufficient reliability and validity in the literature [13].

The purpose of this cross-sectional study was to evaluate the morphometric properties of trabecular bone quality using CBCT in periodontal disease at different stages, comparing smokers and non-smokers. To the best of our knowledge, this is the first study to explore the effects of smoking on trabecular bone microarchitecture in periodontitis using CBCT. The null hypothesis was that smoking leads to lower values of DA, BV/TV, and Tb.Th, and higher values of Tb.Sp across different periodontal disease stages.

## Materials and methods

This cross-sectional study was conducted in accordance with the Declaration of Helsinki and was approved by the Ethics Committee of Istanbul Okan University (Approval No. 2024/176-36). Each patient signed the informed consent form for the study. All participants were informed about the purpose, procedures, potential risks, and benefits of the study. Written informed consent was obtained from all individuals before their inclusion in the study. Participation was voluntary, and participants had the right to withdraw at any time without any consequences. The study included patients who presented to the Department of Dentomaxillofacial Radiology, Faculty of Dentistry, Istanbul Okan University, for CBCT between March and September 2024 due to the evaluation of impacted tooth position or dental implant treatment. A sample size of 15 patients per group (90 patients, 180 ROIs) was calculated using the G*Power 3.1.9.2 software (Heinrich‑Heine‑Universität Düsseldorf, Düsseldorf, Germany) (power (1-β) = 0.80, alpha level (α) = 0.05, effect size (f) = 0.4).

Inclusion criteria were systemically healthy individuals aged 18 years or older with the presence of mandibular molars and mandibular anterior teeth, no pathologies affecting the alveolar bone, and no history of orthodontic treatment, medication use, or radiation/chemotherapy treatment. The selected teeth for measurement (mandibular first molars and central incisors) were required to be free from caries, crowns, root canal treatments, and endodontic lesions. Exclusion criteria included signs of occlusal trauma, bruxism, and patients in the menopausal period.

Smoking status was categorized as follows: the smoking group included patients who smoked over ten cigarettes daily for more than five years, while non-smokers had no cigarette use history. E-cigarette smokers were excluded.

Participants who consented to the study and met the inclusion criteria were grouped based on the stages of periodontitis and gingivitis and their smoking status: smokers with gingivitis, smokers with mild to moderate periodontitis, smokers with severe periodontitis, non-smokers with gingivitis, non-smokers with mild to moderate periodontitis, and non-smokers with severe periodontitis. Participants exhibiting periodontitis stages I and II were classified as mild to moderate periodontitis, while those presenting with stages III and IV were categorized as severe periodontitis. In total, the analysis comprised 90 patients (15 in each group) and included 180 ROIs, with 90 ROIs from the first mandibular molars representing the posterior ROIs and 90 ROIs from the central incisor teeth representing the anterior ROI.

The collected data encompassed demographic information, including age and gender, and periodontal clinical parameters: plaque index (PI), gingival index (GI), periodontal pocket depth (PPD), clinical attachment level (CAL), and bleeding on probing (BOP). A single calibrated operator (I.C.) performed the periodontal assessment utilizing an intraoral mirror and William’s periodontal probe. The periodontal diagnoses of the participants were established in accordance with the 2017 Classification of Periodontal Diseases. Gingivitis was diagnosed based on the presence of bleeding on probing in at least 10% of all assessed sites and the absence of attachment and bone loss. Patients with periodontitis were identified by interdental clinical attachment loss in at least two non-adjacent teeth or the presence of >3 mm periodontal pockets on the buccal surfaces along with ≥3 mm clinical attachment loss. The classification of periodontitis stages was primarily based on CAL, radiographic bone loss, and tooth loss criteria. Specifically, CAL of 1-2 mm was classified as periodontitis stage I, 3-4 mm as periodontitis stage II, and ≥5 mm as stages III-IV. The criteria for tooth loss included no teeth lost for stages I and II, up to four teeth lost for stage III, and five or more teeth lost due to periodontitis for stage IV. Radiographic bone loss in periodontitis stages I and II was confined to the coronal third, while in stages III and IV it extended to the middle or apical third of the root [14].

All CBCT imaging conducted in this study utilized the following exposure parameters: 96 kVp, 5.6 milliampere-seconds, an exposure time of 12 seconds, a voxel size of 200 µm, and a field of view of 13 x 5.5 mm. CBCT scans of individuals meeting the inclusion criteria were recorded without patient information using the Romexis imaging software (Planmeca Romexis, Helsinki, Finland) and then transferred to ImageJ software (National Institutes of Health, Bethesda, Maryland). The microarchitecture of trabecular bone was analyzed by measuring BMPs using the BoneJ plugin of the software. Anterior and posterior ROIs of standard dimensions (5 x 5 x 10 mm) were defined, with the upper surface of each rectangular prism starting just below the apices of the specified teeth (periapical), including the mandibular first molars and the two adjacent central incisors. During ROI selection, the presence of anatomical structures such as the genial tubercle and lingual foramen in the anterior region, and the inferior alveolar canal in the posterior region, served as limiting factors [15]. When these structures were encountered, the CBCT scans were excluded to maintain standardized ROI selection [16]. All measurements were conducted by a single calibrated operator (C.B., with over 10 years of experience in maxillofacial radiology) to ensure consistency and reduce variability. To assess intraoperator reliability, the intraclass correlation coefficient (ICC) was calculated based on 20% of the data that was randomly selected and re-evaluated by the same operator.

**Figure 1:**
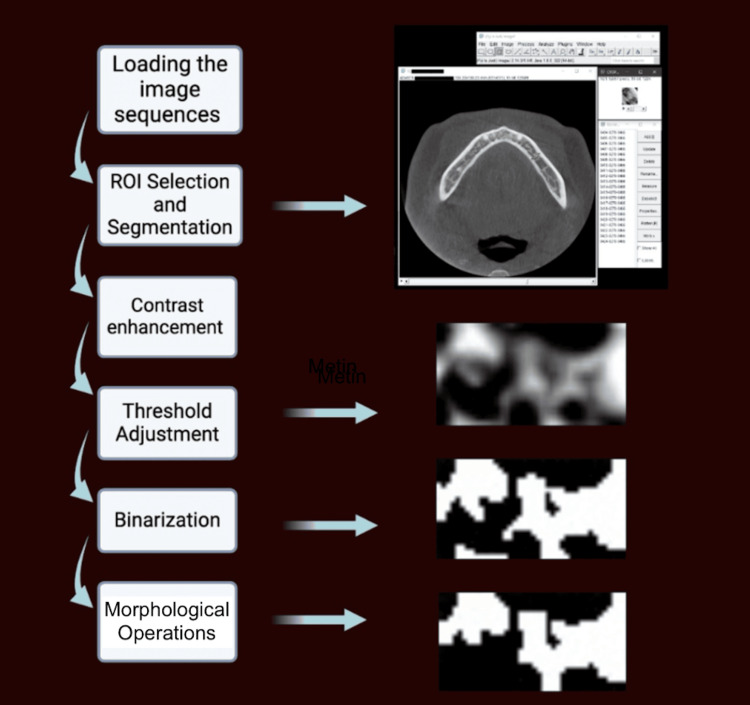
Image processing steps involved in measuring trabecular bone parameters. The ROI selection and segmentation steps include multiple axial slice selections and cropping of the area of interest.

The image processing steps in ImageJ using the BoneJ plugin are summarized in Figure 1. The process began with loading image sequences and converting them from 16-bit to 8-bit for compatibility. Rectangular ROIs were carefully selected and segmented from the entire image sequence. Image contrast was enhanced, and background subtraction was applied to improve the distinction between bone and background. Using the percentile method, a threshold was set to isolate bone structures accurately. Following the binarization of the images, morphological operations such as opening and dilation were employed to refine structural clarity. Once a measurable ROI was obtained, BMP measurements were performed using the BoneJ plugin. Based on the study of Bouxsein et al. [12], measurements were subsequently conducted, focusing on DA, BV/TV, Tb.Th, and Tb.Sp to provide a detailed quantitative analysis of bone morphology.

DA assesses the directional characteristics of trabecular bone. A DA value of 1 indicates an isotropic structure, while values above 1 indicate anisotropy. DA is calculated as the ratio of the longest mean intercept length vector to the shortest.

BV/TV represents the ratio of bone volume to total volume within the ROI, and BV/TV provides an indication of bone density.

Tb.Th indicates the average thickness of the trabeculae, obtained through direct three-dimensional assessment methods.

Tb.Sp represents the mean distance between adjacent trabeculae, and this parameter is also assessed using three-dimensional techniques.

Descriptive statistics for each variable were calculated and presented as “Mean ± Standard Error of Mean” or “Median (Minimum-Maximum).” Before performing the statistical analysis, data were examined with the Shapiro-Wilk test for normality and the Levene test for homogeneity of variances as parametric test assumptions. Student's t-test was used to compare the radiological measurements according to gender, taking into account the fulfillment of parametric test assumptions. After examining test assumptions, a one-way analysis of variance (ANOVA) or Kruskal-Wallis tests were used to evaluate the difference in radiological and clinical measurements between patient groups, followed by Tukey or Dunn’s Bonferroni post hoc tests, respectively. The Spearman correlation coefficient was used to examine the relationship between variables. P <0.05 was considered statistically significant. IBM SPSS Statistics for Windows, Version 21 (Released 2012; IBM Corp., Armonk, New York) was used for the statistical analysis.

## Results

A total of 90 participants, comprising 48 females and 42 males, were examined in this study, focusing on 180 ROIs of the posterior and anterior areas. The average age of participants was 47.07±13.74 years (female: 45.31±14.57; male: 49.07±12.72), with age or gender differences (p<0.001) observed among the groups. The intraclass correlation coefficient (ICC) for quantitative analysis of bone morphology was 0.914, indicating nearly excellent reliability.

Clinical periodontal parameters

Table 1 presents the differences in PI, GI, BOP, PPD, and CAL across groups. The highest PI values were observed in the severe periodontitis groups, as non-smokers and smokers, with measurements significantly differing from other patient groups (Kruskal-Wallis test, H(5) = 26.77; p <0.001). The highest GI value was recorded in non-smokers with severe periodontitis, while the lowest GI was observed in smokers with gingivitis (Kruskal-Wallis test, H(5) = 42.08; p <0.001). In addition, the highest BOP percentage was found in non-smokers with severe periodontitis (One-way ANOVA; F(1,178) = 216.3; p <0.001).

**Table 1:** Periodontal clinical parameters across patient groups. Different superscript letters (a, b, c) in the same column for each variable represent statistical significance (p < 0.05). Test statistics are reported in the text. *Kruskal-Wallis test **One-way ANOVA SEM: Standard error of mean; NA: not available, MP: mild periodontitis, SP: severe periodontitis.

Groups	n	PI*	GI*	BOP (%)**	PD (mm)*	CAL (mm)*
		Mean ± SEM	Mean ± SEM	Mean ± SEM	Mean ± SEM	Mean ± SEM
Gingivitis – smokers	15	1.78 ± 0.1^b^	1.15 ± 0.06^c^	21.48 ± 2.68^c^	2.04 ± 0.1^b^	NA
Gingivitis – non-smokers	15	1.59 ± 0.11^b^	1.36 ± 0.08^bc^	48.83 ± 4.47^b^	1.92 ± 0.05^b^	NA
MP – smokers	15	1.68 ± 0.06^b^	1.22 ± 0.08^bc^	19 ± 3.11^c^	2.33 ± 0.13^b^	1.16 ± 0.14^b^
MP – non-smokers	15	1.83 ± 0.06^b^	1.49 ± 0.06^b^	50.21 ± 4.5^b^	2.45 ± 0.12^b^	1.53 ± 0.16^b^
SP – smokers	15	2.29 ± 0.11^a^	1.45 ± 0.06^bc^	32.18 ± 2.98^c^	3.69 ± 0.2^a^	4.88 ± 0.41^a^
SP – non-smokers	15	2.51 ± 0.11^a^	2.12 ± 0.12^a^	77.03 ± 4.04^a^	3.78 ± 0.32^a^	4.71 ± 0.51^a^
p-value		<0.001	<0.001	<0.001	<0.001	<0.001
Test statistics		H(5) = 26.77	H(5) = 42.08	F(1,178) =216.3	H(5) = 30.25	H(5) = 66.34

Trabecular bone morphometric parameters by group

The analysis of the data in Table 2 revealed no statistically significant difference between the BMPs in the posterior and anterior regions according to the patient groups. In the total patient population, regardless of gender, no statistically significant differences in trabecular microstructure parameters were observed between smokers and non-smokers (one-way ANOVA, F(89,90) ranges between 0.14-0.96, p>0.05).

**Table 2:** Bone morphometric parameters across patient groups. *One-way ANOVA was used for each parameter; p-values and F-statistics (F(df)) are reported in the first row of each parameter block. Groups are categorized as Gingivitis, Mild Periodontitis (MP), and Severe Periodontitis (SP) with smoker and non-smoker subgroups. SEM: standard error of mean, MP: mild periodontitis, SP: severe periodontitis.

BMP	Groups	Smokers			Non-smokers		
		Gingivitis	MP	SP	Gingivitis	MP	SP
Anterior DA	Mean ± SEM	0.56 ± 0.05	0.5 ± 0.06	0.61 ± 0.04	0.56 ± 0.05	0.5 ± 0.06	0.61 ± 0.04
	P	0.126			0.126		
	F*	F(89,90)= 0.96			F(89,90)= 0.96		
Anterior BV/TV	Mean ± SEM	0.45 ± 0.01	0.45 ± 0.01	0.45 ± 0.01	0.45 ± 0.01	0.45 ± 0.01	0.45 ± 0.01
	P	0.459			0.459		
	F*	F(89,90)= 0.23			F(89,90)= 0.23		
Anterior Tb.Th	Mean ± SEM	9.37 ± 0.5	9.49 ± 0.77	8.56 ± 0.74	9.37 ± 0.5	9.49 ± 0.77	8.56 ± 0.74
	P	0.47			0.47		
	F*	F(89,90)= 0.3			F(89,90)= 0.3		
Anterior Tb.Sp	Mean ± SEM	11.12 ± 0.53	10.88 ± 0.89	10.55 ± 0.55	11.12 ± 0.53	10.88 ± 0.89	10.55 ± 0.55
	P	0.59			0.59		
	F*	F(89,90)= 0.18			F(89,90)= 0.18		
Posterior DA	Mean ± SEM	0.54 ± 0.04	0.49 ± 0.05	0.58 ± 0.03	0.54 ± 0.04	0.49 ± 0.05	0.58 ± 0.03
	P	0.736			0.736		
	F*	F(89,90)= 0.25			F(89,90)= 0.25		
Posterior BV/TV	Mean ± SEM	0.44 ± 0.03	0.44 ± 0.03	0.44 ± 0.03	0.44 ± 0.03	0.44 ± 0.03	0.44 ± 0.03
	P	0.999			0.999		
	F*	F(89,90)= 0.24			F(89,90)= 0.24		
Posterior Tb.Th	Mean ± SEM	13.57 ± 1	12.42 ± 0.76	12.17 ± 0.75	13.57 ± 1	12.42 ± 0.76	12.17 ± 0.75
	P	0.33			0.33		
	F*	F(89,90)= 0.16			F(89,90)= 0.16		
Posterior Tb.Sp	Mean ± SEM	11.12 ± 0.53	10.88 ± 0.89	10.55 ± 0.55	11.12 ± 0.53	10.88 ± 0.89	10.55 ± 0.55
	P	0.08			0.08		
	F*	F(89,90)= 0.14			F(89,90)= 0.14		

A statistically significant positive association was observed between posterior BV/TV and mean CAL (Spearman correlation coefficient test, r = 0.261, p = 0.044). The CAL of the first molars demonstrated a positive correlation with posterior area DA (r = 0.125, p = 0.342), BV/TV (r = 0.021, p = 0.871), and Tb.Th (r = 0.109, p = 0.406) measurements, whereas a negative correlation was observed with Tb.Sp values (r = -0.206, p = 0.115) (Table 3).

**Table 3:** Correlation of BMPs with mean CAL and first molar CAL (CAL-Molar). *Spearman correlation coefficient test, r: Spearman’s rho. Test statistics are reported in the text. BV/TV: bone volume fraction, Tb.Th: trabecular thickness, Tb.Sp: trabecular separation, CAL: clinical attachment loss, DA: degree of anisotropy, BMPs: bone morphogenic parameters.

Variables	CAL (mm), r (p-value)	CAL-Molar (mm), r (p-value)
Anterior DA	0.139 (0.289)	
Anterior BV/TV	0.061 (0.643)	
Anterior Tb.Th	-0.119 (0.364)	
Anterior Tb.Sp	0.031 (0.813)	
Posterior DA	0.016 (0.904)	0.125 (0.342)
Posterior BV/TV	0.261 (0.044)	0.021 (0.871)
Posterior TbTh	-0.027 (0.836)	0.109 (0.406)
Posterior TbSp	-0.216 (0.098)	-0.206 (0.115)

Gender and smoking effects on trabecular bone morphology

BMP measurements showed statistically significant differences among genders. Table 4 demonstrates that smoking had no effect on trabecular bone parameters in either females or males (Student’s t-test, p >0.05).

**Table 4:** Comparison of BMPs measurements by gender and smoking status. *Student's t-test was used for each gender and smoking status comparison. Each parameter is listed separately under Anterior and Posterior regions with subgroup analysis by gender and smoking status. SEM: Standard error of mean, BMP: bone morphogenic parameter.

BMP			DA			BV/TV			Tb.Th			Tb.Sp		
Region	Gender	Smoking status	Mean ± SEM	P	t*	Mean ± SEM	P	t*	Mean ± SEM	P	t*	Mean ± SEM	P	t*
Anterior	Female	Smoker	0.57 ± 0.04	0.052	t(27)=-0.99	0.45 ± 0.007	0.944	t(34)= 0.24	9.53 ± 0.56	0.452	t(42)= -0.7	10.84 ± 0.70	0.423	t(54)= -0.64
		Non-smoker	0.47 ± 0.15			0.44 ± 0.013			10.28 ± 0.73			11.56 ± 0.55		
	Male	Smoker	0.54 ± 0.04			0.44 ± 0.008			8.82 ± 0.53			10.85 ± 0.41		
		Non-smoker	0.47 ± 0.35			0.45 ± 0.007			8.84 ± 0.87			10.63 ± 0.67		
Posterior	Female	Smoker	0.53 ± 0.3	0.632	t(16)=-2.06	0.42 ± 0.03	0.342	t(39)= -0.97	13.92 ± 0.68	0.903	t(27)= -0.02	14.76 ± 1.07	0.215	t(27)= -0.26
		Non-smoker	0.56 ± 0.03			0.45 ± 0.01			13.79 ± 0.84			16.52 ± 0.90		
	Male	Smoker	0.53 ± 0.03			0.45 ± 0.01			11.75 ± 0.48			13.95 ± 0.60		
		Non-smoker	0.50 ± 0.03			0.42 ± 0.03			12.63 ± 0.97			15.26 ± 1.12		

## Discussion

The evaluation of alveolar bone can be conducted through various methods, including clinical examination, histological assessment, periapical and panoramic radiographs, CBCT, micro-CT, and digital analysis. The comprehensive assessment combining clinical periodontal parameters with advanced imaging techniques can improve diagnostic accuracy, identify risk factors, and impact treatment planning for periodontal disease. Accordingly, the purpose of this study is to assess the morphometric properties of trabecular bone quality in periodontal disease at different stages, comparing smokers and non-smokers using CBCT. The current study found no statistically significant differences in BMPs based on periodontal condition or smoking status. Notably, a significant positive association was observed between posterior BV/TV and mean CAL. In the first molars, CAL was positively correlated with posterior area DA, BV/TV, and Tb.Th, while a negative correlation was observed with Tb.Sp. Consequently, the null hypothesis that smoking leads to lower DA, BV/TV, and Tb.Th values and higher Tb.Sp values in relation to periodontal disease stages was rejected.

Trabecular bone microarchitecture is a crucial indicator for assessing the mechanical properties of trabecular bone, as it directly influences the bone's strength, stiffness, and ability to withstand loads. Numerous parameters are essential when evaluating trabecular bone microarchitecture, including BV/TV, TbTh, TbSp, TbN, bone surface, bone surface density, connectivity density, structure model index, degree of anisotropy, and mean intercept length [12, 17]. According to the American Society for Bone and Mineral Research guidelines, BV/TV, Tb.N, Tb.Th, and Tb.Sp are the essential variables to report for trabecular bone morphology [12]. These parameters play a critical role in assessing the mechanical properties and health of trabecular bone. However, there is no study in the literature that specifically analyzes trabecular bone microarchitecture in relation to smoking and periodontal disease. This study will serve as a significant contribution to the literature in this area.

Various imaging modalities, including conventional radiographs, are utilized to assess trabecular alveolar bone quality. Conventional radiographs identify general bone loss and trabecular architecture changes, but they have limitations such as their 2D nature, geometric distortion, and superimposition [18]. Fractal analysis (FA) has been applied to panoramic [19, 20] and periapical [21, 22] radiographs, with studies showing no difference in trabecular quality between severe and mild-to-moderate periodontitis, although FA values were lower in periodontitis than in gingivitis [23, 24]. However, some studies found a notable reduction in FA values as the stages of periodontitis progressed [25]. The absence of CBCT in previous research limits the accurate assessment of alveolar bone morphology. This study is the first to analyze trabecular bone characteristics in 3D by assessing BMPs across different stages of periodontitis and gingivitis with CBCT.

**Figure 2:**
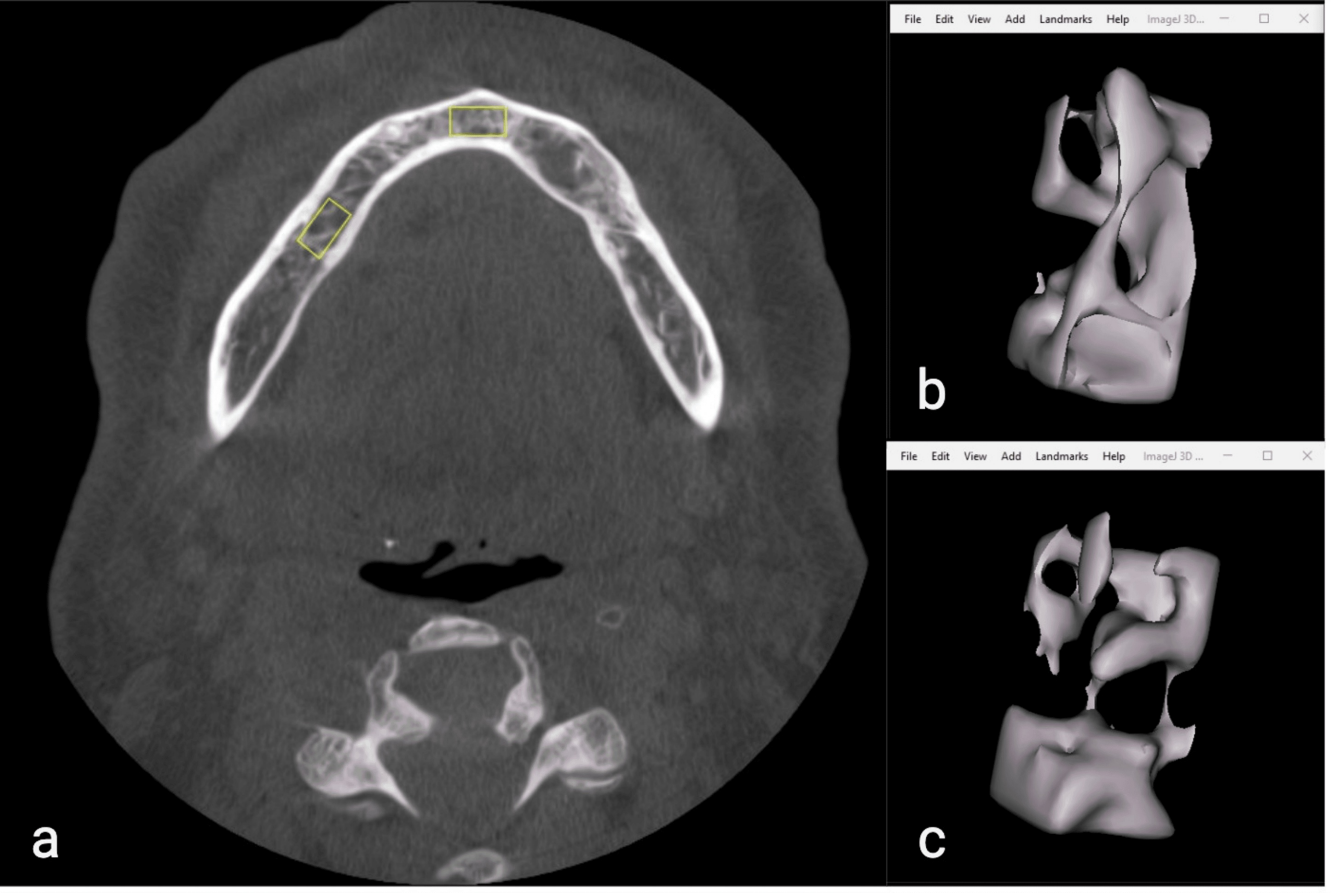
Sselection of the anterior and posterior ROIs (a), a segmented anterior trabecular bone (b), and a segmented posterior trabecular bone (c). The image is derived from a patient within the study population and is shared with the patient’s informed consent.

While CBCT is commonly used in dentistry to assess bone quality, its grey value accuracy has limited quantitative analysis. Recently, studies have suggested that bone microarchitecture analysis, traditionally performed with micro-CT, could be adapted for CBCT in clinical applications, provided that parameter values remain consistent across different scanning settings [13, 26-28]. A morphometric analysis indicated a strong correlation between BV/TV values from CBCT images and CT values from multislice CT, supporting the use of trabecular bone morphometry to assess mandibular cancellous bone density [29]. Previous studies indicate that voxel size can significantly affect bone volume measurements, particularly in porous bone, while kV and mA values generally have minimal impact. Notably, variations in bone volume are observed when voxel size exceeds 200 µm [26, 30]. In this study, we used a standardized protocol, including a voxel size of 200 µm along with other consistent parameters, to optimize measurement consistency and minimize potential variability. Furthermore, the rationale for selecting the ROIs was to represent the trabecular architecture while ensuring that sufficient bone tissue (5 x 5 x 10 mm) was present in both the posterior and anterior areas, which are most affected by periodontal changes. Choosing interdental or furcal regions could lead to unstandardized ROIs and result in measurement variations. Therefore, the apices of the first mandibular molar and central incisors were specifically selected to investigate the local and systemic effects of smoking on alveolar trabecular bone (Figure 2).

Several studies have documented the detrimental effects of smoking on bone loss. Smoking induces an imbalance in the RANKL/OPG ratio, leading to increased bone resorption and accelerating bone turnover in the alveolar bone. Additionally, smoking can increase oxidative stress, making bone tissue more susceptible to damage [6, 31-33]. Bergström [33] assessed the long-term effects of chronic smoking on periodontal bone height by measuring the CEJ-PBC (cementoenamel junction-periodontal bone crest) distance on bite-wing radiographs of premolars. Smokers showed a significantly greater baseline CEJ-PBC distance and experienced a 10-year bone height reduction of 0.74 mm compared to 0.27 mm in non-smokers, indicating an accelerated reduction rate due to smoking. In the evaluation of distal radius microstructure and macrostructure differences between smokers and non-smokers, Johnson et al. [34] examined distal radius microstructure and macrostructure in smokers versus non-smokers, adjusting for age and BMI. They found that smokers had 33% higher cortical porosity with reduced cortical density, leading to lower overall bone density. However, trabecular parameters (BV/TV, TbTh, TbN, TbS) and macro-scale characteristics (bone volume, bone mineral content, volumetric bone mineral density) were similar between smokers and non-smokers. In the study by Shah et al. [35], the alveolar bone microstructure values obtained using micro-CT from biopsies taken at post-extraction sites revealed similar mineralization profiles in smokers and non-smokers, consistent with our results. The stability of the mineralized matrix in bone may protect bone quality from the adverse effects associated with smoking. Future research that includes examinations of cortical bone indices, along with trabecular microarchitecture assessments, could enhance our understanding of the changes related to smoking in the jaws.

Our study found a significant positive correlation between posterior BV/TV and mean CAL. In the first molars, CAL was positively correlated with posterior DA, BV/TV, and Tb.Th, while Tb.Sp showed a tendency to decrease. These findings support the concept of buttressing bone formation, which occurs in response to bone resorption in areas adjacent to active inflammation, strengthening the remaining bone [36, 37]. This phenomenon has been observed in experimental models of periodontal bone loss in animals and is less apparent in humans but supported by histometric studies [37, 38]. While this research may deepen our understanding of trabecular bone dynamics in relation to periodontal health, future studies assessing trabecular bone quality across various populations could shed light on the mechanisms of buttressing bone formation.

In the study by Agarwal et al. [39], musculoskeletal health was assessed through dual-energy X-ray absorptiometry (DXA), high-resolution peripheral quantitative computed tomography (HR-pQCT), trabecular bone score, and vertebral fracture assessment in both current and past smokers as well as nonsmokers. The findings indicated that current smoking is associated with trabecular deterioration in the spine and peripheral skeleton in men, while women experience cortical deficits, suggesting that smoking may have gender-specific skeletal effects. Estrogen is crucial for maintaining bone health, mainly by inhibiting bone resorption. Its deficiency is well-established as a factor in the development of osteoporosis, particularly in postmenopausal women and men with low estradiol levels. Smokers tend to have reduced urinary excretion of estradiol and estriol during the luteal phase, as well as a diminished serum response to oral estrogen, resulting in a state of estrogen deficiency that negatively impacts bone health [40]. Conversely, some studies suggest that sex has little to no impact on trabecular bone architecture and its age-related changes [41-43]. Furthermore, smoking did not notably affect BMPs within either gender group. The lack of significant differences between males and females in BMPs may be related to the average age of our female participants, who are in the premenopausal phase, potentially mitigating the effects of estrogen deficiency on bone. Importantly, the absence of marked gender and age disparities within our groups enables a clearer analysis of the relationship between smoking and periodontal disease, helping isolate the effects of smoking on bone health without the confounding factor of hormonal changes related to menopause.

This study has several limitations. A limitation is the cross-sectional design, which restricts the ability to establish causal relationships between periodontal disease stages and trabecular bone changes. In addition, although patients with systemic diseases, on medications, or in the menopausal period were excluded, the study’s limitations include the lack of biochemical and genetic assessments of factors such as osteoporosis and hormonal influences. Future research should include patients with systemic conditions such as osteopenia, osteoporosis, and diabetes to better assess the relationship between these conditions and trabecular bone quality.

## Conclusions

The results of this study indicate that in systemically healthy individuals, smoking status, periodontal diagnosis, age, and gender did not significantly influence alveolar trabecular bone microarchitecture as assessed by CBCT. However, the significant correlation between posterior BV/TV and CAL suggests a localized relationship between periodontal tissue breakdown and bone quality, particularly in the posterior region. Additionally, the observed trend toward increased trabecular thickness with greater attachment loss may reflect compensatory or disease-related changes in trabecular bone structure. These findings highlight the importance of considering site-specific bone changes in the context of periodontal disease progression. Further longitudinal and histological studies are warranted to elucidate the biological mechanisms underlying these associations.
